# Metastatic urothelial carcinoma of the urinary bladder in a Sumatran tiger (*Panthera tigris sondaica*)

**DOI:** 10.1002/vms3.771

**Published:** 2022-03-03

**Authors:** Louise van der Weyden, Clare Tibbs, Chris Knott, Melanie Dobromylskyj

**Affiliations:** ^1^ Sanger Institute Wellcome Genome Campus Hinxton Cambridge UK; ^2^ Tibbs and Simmons Farm Animal Veterinary Surgeons Redhill Bristol UK; ^3^ Finn Pathologists Histopathology Department Diss Norfolk UK

**Keywords:** Panthera tigris, Sumatran tiger, tiger, transitional cell carcinoma, urinary bladder, urothelial cancer

## Abstract

A 15‐year‐old spayed female Sumatran tiger (*Panthera tigris sondaica*) was presented with a short history of haematuria and dysuria, non‐responsive to antibiotics, and a gradual decline to inappetence over a period of 2–3 months. Ultrasound examination showed a thickened urinary bladder wall and the renal pelvis of right kidney was dilated and cystic. A presumptive diagnosis of renal failure was made, and the tigress was euthanised due to deteriorating quality of life and pronounced weight loss. Histopathology revealed extensive erosion of the urinary bladder wall and marked congestion of the submucosal vasculature, a potential cause of the haematuria observed clinically. Numerous foci of neoplastic cells were also observed throughout the lung parenchyma as well as within lymphatic vessels of the lung, the liver and the kidney. A diagnosis of a metastatic non‐papillary high‐grade urothelial carcinoma (UC) of the urinary bladder was made. Consistent with this diagnosis, immunohistochemistry revealed the neoplastic cells were negative for uroplakin III, as has been reported for a subset of high‐grade, infiltrative urinary bladder UCs of canines and humans. This is the first report of a primary tumour of the urinary bladder in a tiger and the first report of UC in a tiger.

## Graphical abstract

This is the first report of a primary tumour of the urinary bladder of a tiger and the first report of urothelial carcinoma in a tiger. Autopsy revealed neoplastic tissue was present throughout the urinary bladder wall and lymphatic vessels, and deposits of neoplastic cells were also seen within the lungs, kidney and the liver. We believe this report will be of great interest to both veterinary and comparative pathologists and oncologists alike.

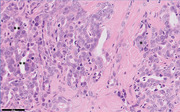

## 1 INTRODUCTION

Urothelial carcinoma (UC; formerly known as transitional cell carcinoma) is the most common type of bladder cancer in dogs and cats, although the incidence in cats is comparatively low (∼2% versus 0.38–0.56% of all malignancies, respectively) (Meuten & Meuten, 2017; Shida et al., 2010; Wimberly & Lewis, 1979). UC has rarely been reported in non‐domesticated felids, with cases mentioned only in captive‐bred fishing cats (Prionailurus viverrinus) (Sutherland‐Smith et al., 2004), a Pallas’ cat (*Otocolobus manul*) and a lion (*Panthera leo*) (Moresco et al., 2020). In a recent study of neoplasia in captive *Panthera* species, which included 70 tigers and a total of 108 neoplasms, there was only one report of a neoplasia in the urinary system, specifically a renal papillary adenoma (Kloft et al., 2019). In this case report, we present a primary non‐papillary high‐grade metastatic UC of the urinary bladder in a Sumatran tiger.

## 2 CASE PRESENTATION

A captive 15‐year‐old spayed female Sumatran tiger (*Panthera tigris sondaica*) residing within a zoological collection in the United Kingdom and on long‐term medication for presumed arthritis (meloxicam, Rheumocam, Chanelle Pharma, Co Galway, Ireland; titrated to effect, q24h, per os, 0.05–0.1 mg/kg), was presented with a one week history of haematuria and dysuria, along with reduced appetite over a period of 2–3 months. Antibiotics were administered per os for a presumed bacterial cystitis (12.5 mg/kg BID; amoxicillin and clavulanic acid, Synulox Bolus, Zoetis, New Jersey, USA), followed 10 days later by per os robenacoxib (1 mg/kg SID; Onsior, Elanco, Liverpool, UK) and tramadol (1–2 mg/kg) as the keepers reported that she appeared nauseous. However, the following day she refused food, thus cefovecin was administered by dart (8 mg/kg, Convenia; Zoetis). Urinalysis revealed haematuria, pyuria and proteinuria (dipstick analysis). Two weeks later, the tigress displayed polydipsia, muscle mass loss and weakness. Repeated urinalysis revealed similar abnormalities, and the treatment with cefovecin was repeated. Three days later, urinalysis showed no change, and the tigress was refusing food except for a synthetic diet used for critically ill carnivores (Emeraid IC Carnivore, EmerAid, Illinois, USA) with a Vitamin B_12_ supplement (ViteBee, Dechra, Northwich, United Kingdom).

Three days later, the tigress was anaesthetised with intramuscular administration of medetomidine (80 μg medetomidine/kg bodyweight) and ketamine (5 mg ketamine/kg bodyweight) via dart to allow for a veterinary examination. There were no abnormalities detected of the skin or the head, apart from pale mucous membranes. Placement of a urinary catheter was attempted; however, it was unsuccessful, thus raising concern for urethral obstruction. On ultrasound examination, a thickened bladder wall, left renomegaly and right kidney pyelectasia containing a large volume of fluid associated with marked compression and thinning of the surrounding renal parenchyma were observed. A presumptive diagnosis of renal failure was made, and blood samples were obtained for confirmation (urea = 133.0 mmol/L; creatinine = 2695 μmol/L; total protein high = 85.0 g/L, albumin low = 23.2 g/L). Due to poor quality of life and a poor prognosis, the tigress was humanely destroyed with a free bullet while anaesthetised.

At post‐mortem, the bladder did not contain any urine and the bladder wall was thickened and inflamed. There was major loss of muscle mass with a generalised absence of subcutaneous fat (although abdominal fat was still present). Within the stomach there was an absence of ingesta and no mucosal ulcers were noted. Ingesta was also absent within the small intestine; however, faeces was present in the large intestine. Gross lesions were noted within the right kidney, urinary bladder wall and apical lung lobes. Samples from the lesions on the kidney, urinary bladder wall and apical lung lobes were taken for histological analysis. The liver showed no gross abnormalities; however, a representative sample was also taken for histological analysis.

**FIGURE 1:**
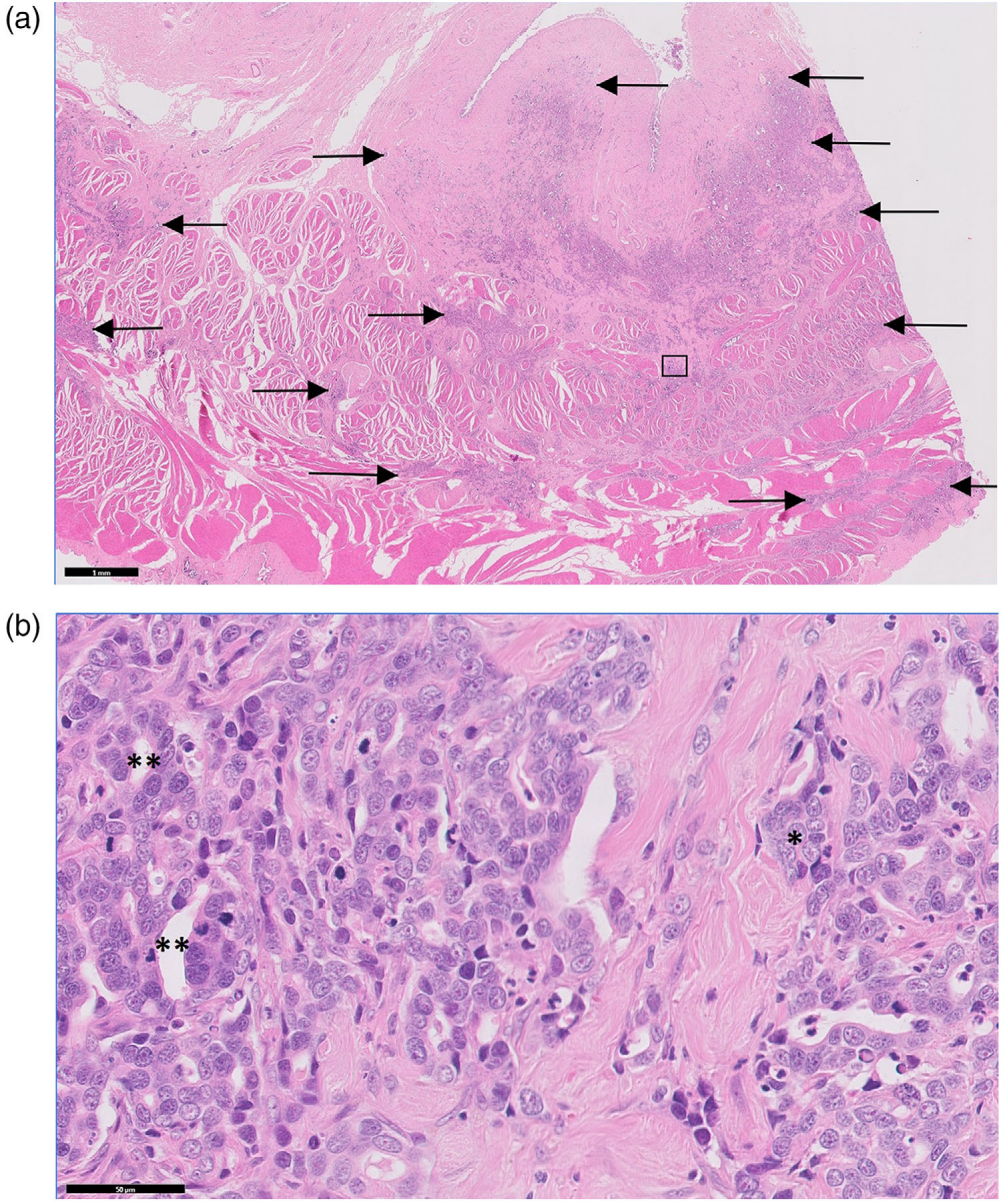
Histopathology of a urinary bladder lesion in a Sumatran tiger. (a) Low power magnification showing neoplastic epithelial cells present throughout the bladder wall, extending from the submucosa, through the tunica muscularis to the serosa. Neoplastic infiltrates indicated by black arrows. Box indicates area represented at higher power magnification in b. 10× magnification and scale bar = 1 mm, haematoxylin and eosin stain. (b) High power view of area indicated by the box in a. Tumour cells were mostly cuboidal in shape and arranged in solid nests and packets (*) or tubular structures (**). 400× magnification and scale bar = 50 μm, haematoxylin and eosin stain

Following fixation in 10% buffered formalin and embedding in paraffin wax, 4‐μm tissue sections were cut and routinely stained with haematoxylin and eosin (HE). In all four sections examined from the urinary bladder, neoplastic epithelial cells were present throughout the bladder wall, extending from the tunica submucosa, through the tunica muscularis to the tunica serosa (Figure 1a), with neoplastic aggregates noted within many lymphatic vessels. The tumour cells were mostly cuboidal to columnar in shape and arranged in tubular and papillary structures, lacking any obvious ciliation, although in some fields the growth pattern was more solid and anaplastic (Figure 1b). There was moderate nuclear atypia, with a mitotic count of 66 per 10 high power fields (400×; 2.37 mm^2^). There was a pronounced desmoplastic response to the neoplastic infiltration, especially within the tunica muscularis. The lining epithelium of the bladder had extensively eroded, and the submucosal vasculature was markedly congested, potentially the cause of the haematuria seen clinically. In one of the sections, remnants of the mucosal urothelial lining were proliferative, but not definitively neoplastic; however, neoplastic aggregates were present within 200 μm of the area.

**FIGURE 2:**
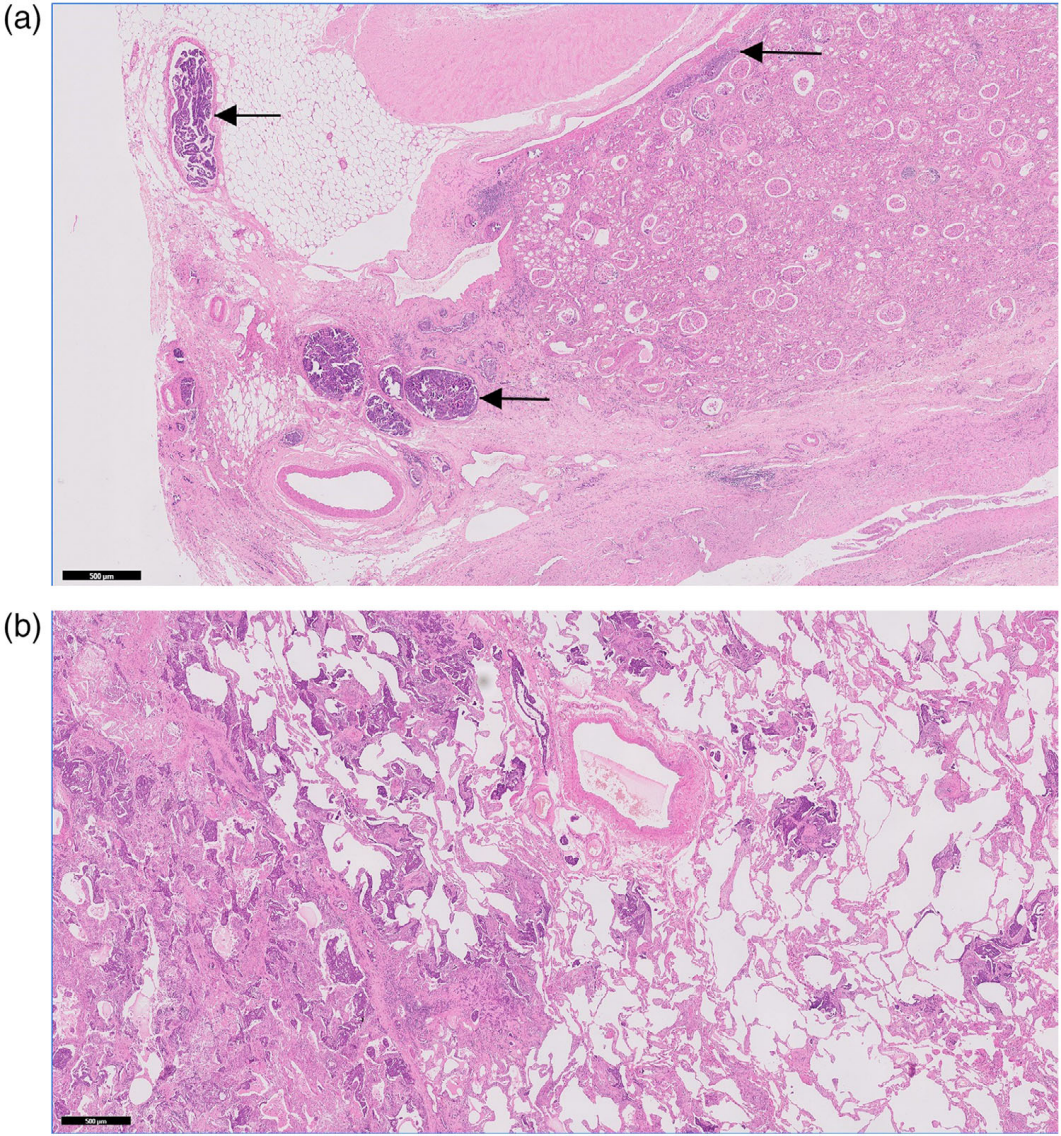
Histopathology of metastatic lesions in a Sumatran tiger. (a) Substantial aggregates of neoplastic epithelial cells seen in the lymphatics of the kidney (black arrows); 20× magnification and scale bar = 500 μm. Haematoxylin and eosin stain. (b) Numerous aggregates of neoplastic epithelial cells seen in the lung; 20× magnification and scale bar = 500 μm. Haematoxylin and eosin stain

Similar neoplastic cells were also present forming clusters and aggregates within parts of the kidney (Figure 2a), liver and lungs (Figure 2b), with further evidence of widespread lymphatic invasion. The renal vasculature was moderately congested, with widespread mild tubular dilatation and patchy, mild‐to‐moderate interstitial fibrosis sometimes associated with a mild, predominantly lymphocytic inflammatory infiltrate. Some glomeruli appeared shrunken, and Bowman's capsules were variably thickened, with occasional mineralisation and fibrous crescent formation. Several variably sized foci of neoplastic cells were also present within the liver, forming irregular tubular structures or smaller solid clusters, sometimes within lymphatic vessels. The lung parenchyma also contained numerous foci of neoplastic cells scattered haphazardly throughout, sometimes within lymphatic vessels.

A diagnosis of urinary bladder carcinoma metastatic to lung, liver and kidney was made. The gross and histolopathologic presentation of the case suggested the urinary bladder as the most likely primary site. In an effort to confirm the diagnosis of UC, immunohistochemistry was performed, looking for the urothelial marker uroplakin III (as per Sledge et al., 2015), and, while the surrounding healthy urothelium showed positive staining (confirming suitability of the antibody for use in tiger tissues), the neoplastic cells did not stain positively. Loss of UPIII expression has been reported in high‐grade, infiltrating urinary bladder UCs of both canines and humans (Parker et al., 2003; Ramos‐Vara et al., 2003); therefore, the favoured diagnosis remained a non‐papillary high‐grade UC of the urinary bladder which had metastasised to the other tissues.

## 3 DISCUSSION

In this case report, the neoplastic cells in the urinary bladder were present throughout the entire bladder wall from the submucosa to the serosa and had demonstrated infiltration into the lymphatic vessels consistent with the histological phenotype of highly invasive UCs seen in domestic cats (van der Weyden et al., 2021). The favoured diagnosis was a non‐papillary high‐grade UC of the urinary bladder which had metastasised to the other tissues, as the apparent lack of ciliation made a pulmonary (bronchogenic) origin unlikely, and the relatively small neoplastic aggregates in both the liver and kidney made a hepatic or renal origin also unlikely. Differential diagnoses of mammary carcinoma or apocrine adenocarcinoma from the skin cannot be definitively excluded; however, while there was no gross evidence of any lesions in the mammary glands or skin, it cannot be discounted that a small primary mass may have been present in these tissues.

In domestic cats, UC of the urinary bladder is typically invasive at the time of diagnosis (Brearley et al., 1986; van der Weyden et al., 2021; Wimberly & Lewis, 1979) and thus classified as high‐grade (Meuten & Meuten, 2017), with histological features similar to high‐grade (grade 3) UC of the urinary bladder in humans (Compérat et al., 2019). At the time of diagnosis, the neoplasm in this tiger had metastasised to all of the visceral tissues that were sampled (lung, kidney and liver), with particularly notable secondary growth occurring in the lung. This is consistent with the highly aggressive nature of UC in cats, where studies have reported recurrent lesions (Schwarz et al., 1985; van der Weyden et al., 2021) and evidence of metastasis at the time of diagnosis, typically to the regional lymph nodes or lungs, in 12–50% of cases (Griffin et al., 2020; Schwarz et al., 1985; Sutherland‐Smith et al., 2004). In the bladder, the stromal response to neoplastic cells can show a range of morphologies, including mucinous, myxomatous or desmoplastic (scirrhous) (van der Weyden et al., 2021). In this case report, a prominent desmoplastic response to the neoplastic infiltration was observed, as has been reported in other studies of UC in cats (Patnaik et al., 1986; van der Weyden et al., 2021; Wimberly & Lewis, 1979) and in humans (Cheng et al., 2019).

Immunohistochemical analysis of the expression of UPIII, cytokeratin 7 (CK7) and cytokeratin 20 (CK20) have been commonly used as diagnostic markers of urothelial differentiation in primary tumours and metastases in both humans and canines (Gruver et al., 2012; Ramos‐Vara et al., 2003; Sledge et al., 2015). Uroplakins are a family of membrane‐associated proteins expressed by urothelial cells that are important for cell–cell adhesion and maintenance of water impermeability; forming a complex along the apical membrane of the cells forming the most superficial layer of the urothelium (Wu et al., 2009). The urothelium contains cytokeratins associated with simple‐epithelium type cytokeratins (CK7, 8 and 18–20) and stratified‐epithelium cytokeratins (CK13 and CK17) (Moll et al., 1988). However, it is important to note that unlike UPIII, both CK7 and CK20 are not specific for UC, as they are also expressed in carcinomas of ovarian, pancreatic and cholangiolar origin (Chu et al., 2000). Importantly, studies in humans have reported that some invasive and metastatic UC cases show a lower sensitivity of detection of UPIII (Parker et al., 2003). One study reported that while UPIII is a specific and sensitive marker for canine UCs, negative results may be observed with anaplastic tumours (Ramos‐Vara et al., 2003). UPIII and CK7 positivity has been reported to be lost in some canine high‐grade or anaplastic UCs that invade into the urinary bladder wall, suggesting a lack of differentiation or epithelial‐mesenchymal transition can favour invasion (Sledge et al., 2015). Similarly, loss of UPIII and CK7 expression/reactivity has been reported to associate with an aggressive/invasive phenotype in bovine UCs (Cota et al., 2014) and UPIII expression is negatively correlated with tumour grade in rodent models of chemically induced urinary bladder carcinogenesis (Ogawa et al., 1999). UPIII positivity/expression in feline UCs has not been as extensively investigated as it has in human and canine UCs, with no published case studies using UPIII IHC, although it has been reported to label feline UC (Meuten & Meuten, 2017). However, in correlation with some human, canine, bovine and rodent UCs, loss of UPIII expression may occur with some feline high‐grade infiltrative UCs.

It has been suggested that there may be a genetic predisposition/propensity for the development of neoplasia at the *Panthera* genus (or higher) level, which may be compounded by mutations from in‐breeding in captive populations (Moresco et al., 2020). In a study of 195 cases of neoplasia in 16 species of non‐domesticated felid species living under managed care (80 and 115 cases of *Pathera* and non‐*Panthera* species, respectively), the *Panthera* species had a significantly higher prevalence of neoplasia than non‐*Panthera* species (50% vs. 13%, respectively), with the percentage of malignant neoplasms also significantly higher in the *Panthera* species (86% vs. 61% respectively) (Moresco et al., 2020). The reproductive system (reproductive organs and mammary glands) was the most commonly affected system (21% of cases), followed by the haematolymphoid system (18%) and the respiratory system (16%) (Moresco et al., 2020), with only one case involving the urinary system (UC of the urinary bladder in a lion). Prior to the case presented here, it was the only reported case of UC in a member of the *Panthera* species, consistent with the rarity of reports of UC of the urinary bladder in non‐domestic felids, having been reported in fishing cats (Sutherland‐Smith et al., 2004), and a Pallas’ cat (Moresco et al., 2020), and the relatively low incidence of UC in domestic cats. It has been speculated that the lower incidence of UC of the urinary bladder in domestic cats, relative to dogs and humans, may be due to the lower quantities of tryptophan metabolites present in feline urine (Brown & Price, 1956) and/or potential underdiagnosis, with UCs often occurring in geriatric cats with concurrent diseases (Meuten & Meuten, 2017). Whether these factors play a role in the incidence of UC of the urinary bladder in the *Panthera* species remains to be determined. Survival times of domestic cats with UC of the urinary bladder significantly increase after treatment with partial cystectomy and nonsteroidal anti‐inflammatory drugs (NSAIDs), such as piroxicam, meloxicam or robenacoxib (Griffin et al., 2020). However, the potential benefit of treatment of urinary tract UC in non‐domesticated felids with partial cystectomy and NSAIDs remains to be determined.

In conclusion, this is the first report of a UC in the urinary bladder of a tiger. Furthermore, the presentation of the UC was consistent with that seen in UC of domesticated cats, in that high‐grade UCs appear to be aggressively malignant, with widespread metastasis. It also shows for the first time that uroplakin III IHC can be used in tiger tissues and that, similar to high‐grade, infiltrative UC in canines and humans, loss of UPIII can be observed. It is hoped that further investigations of tigers in zoological facilities or wildlife reserves will add to improved knowledge and understanding of the tumour types developed by this species, paving the way for earlier detection and successful management.

## CONFLICT OF INTEREST

The authors declare no potential conflicts of interest with respect to the research, authorship and/or publication of this article.

## AUTHOR CONTRIBUTIONS

Louise van der Weyden: conceptualisation; writing‐original draft. Clare Tibbs: data curation; writing‐review & editing. Chris Knott: data curation; writing‐review & editing. Melanie Dobromylskyj: conceptualisation; data curation; writing‐review & editing.

## ETHICS STATEMENT

The owner of the tigress gave consent for the case to be written up.

### PEER REVIEW

The peer review history for this article is available at https://publons.com/publon/10.1002/vms3.771.

## Data Availability

The data that support the findings of this study are available from the corresponding author upon reasonable request.
